# Epigenetic Modifications in Thyroid Cancer Cells Restore NIS and Radio-Iodine Uptake and Promote Cell Death

**DOI:** 10.3390/jcm7040061

**Published:** 2018-03-21

**Authors:** Sabine Wächter, Alexander I. Damanakis, Moritz Elxnat, Silvia Roth, Annette Wunderlich, Frederik A. Verburg, Sebastian A. Fellinger, Detlef K. Bartsch, Pietro Di Fazio

**Affiliations:** 1 Department of Visceral Thoracic and Vascular Surgery, Philipps University Marburg, Baldingerstrasse, 35043 Marburg, Germany; seckhard@med.uni-marburg.de (S.W.); alexander.damanakis@me.com (A.I.D.); moritz_elxnat@gmx.de (M.E.); rothsi@med.uni-marburg.de (S.R.); wunderli.an@gmail.com (A.W.); bartsch@med.uni-marburg.de (D.K.B.); 2 Department of Nuclear Medicine, Philipps University Marburg, Baldingerstrasse, 35043 Marburg, Germany; FrederikAnton.Verburg@uk-gm.de (F.A.V.); sebastian.fellinger@med.uni-marburg.de (S.A.F.)

**Keywords:** histone deacetylase inhibitors, thyroid cancer, NIS, H19, miRNAs, TTF1, cell viability, epigenetics

## Abstract

Epigenetic modifications have been identified as being responsible for the de-differentiation of thyroid tissue and its malignant transformation. Cell proliferation inhibitory effects of the pan-deacetylase inhibitors panobinostat, SAHA and Trichostatin A (TSA), the modulation of the sodium iodide symporter (NIS; SLC5A5), thyroid transcription factor 1 (TTF1), high mobility group A2 (HMGA2), and H19 and their putative targeting miRNAs have been evaluated in vitro. The cell viability was measured in five thyroid cancer cell lines (FTC133, TPC1, BCPAP, 8505C, C643) by real time cell analyzer xCELLigence. Expression of the above mentioned markers was performed by RT-qPCR and Western Blot. Radioiodine up-take was detected by Gamma Counter with I^131^. Cell viability decreased after treatment in all five cell lines. 10 nM panobinostat; 1 µM TSA or 10 µM SAHA caused a significant over-expression of NIS transcript in all five cell lines, whereas NIS protein was up-regulated in FTC133, BCPAP, and C643 cell lines only. Radioiodine up-take increased in FTC133 and C643 cells after 48 h of treatment with 10 nM panobinostat and 1 µM TSA. A significant down-regulation of the oncogene HMGA2 was detected in all five cell lines; except for TPC1 cells that were treated with 1 µM TSA. In accordance, hsa-let-7b-5p and hsa-let-7f-5p were stable or significantly over-expressed in all of the cell lines, except for TPC1 cells that were treated with 10 µM SAHA. TTF1 was significantly down-regulated in FTC133, BCPAP, and 8505C cells; whereas, TPC1 and C643 showed an up-regulated or stable expression. TTF1 was over-expressed in samples of human anaplastic thyroid cancer; whereas, it was down-regulated in follicular and undetectable in papillary thyroid cancer. H19 was over-expressed after 48 h treatment, except for BCPAP cells that were treated with panobinostat and SAHA. H19 was differently expressed in human anaplastic, follicular and papillary thyroid tumor samples. Deacetylase inhibitors reduced cell viability, restored NIS and H19, and suppressed the oncogenes HMGA2 and TTF1 in thyroid cancer cells.

## 1. Introduction

Thyroid cancer is the most common endocrine malignancy and its incidence is increasing worldwide every year. Follicular and papillary thyroid cancer (FTC and PTC) has shown a favorable prognosis and low recurrence due to the effectiveness of surgical therapy, followed by I^131^ radio ablation and TSH (thyroid stimulating hormone) suppression with T4. Poorly differentiated (PDTC) and anaplastic thyroid cancer (ATC) are characterized by high metastatic potential and a failure of radioiodine response [1], resulting in poor patient outcome and short survival rate. The resistance to radioiodine in PDTC as well as in ATC is caused by a dysfunction of the sodium-iodide symporter (NIS; SLC5A5). This dysfunction is mainly due to suppression, loss of NIS gene [2,3], and/or a distorted alignment at the cytosolic membrane site [4]. NIS transcript and protein level have been shown to be low in undifferentiated thyroid cancer, the re-expression of NIS gene could restore the radioiodine up-take [5]. Furthermore, it has been shown that NIS is a target of miRNAs belonging to Let7 family. In particular, the expression of hsa-let-7f-5p and hsa-let-7b-5p is significantly high in patients that are affected by thyroid cancer and this is correlated with the strong down-regulation of NIS mRNA [6]. 

Furthermore, it has been shown that HMGA2 (High Mobility Group AT-hook 2), which is a well-known non-transcription factor with oncogenic properties, is highly expressed in several benign tumors of mesenchymal origin, e.g., pituitary adenomas [7,8] and several malignancies, including thyroid cancer [6]. It is responsible for supporting the activity of proliferation related transcription factors, e.g., E2F1, leading to proliferation and survival of tumor cells [9]. HMGA2 down-regulation, as mediated by epigenetic modifications that imply the over-expression of Let7 family miRNAs, leads to cell cycle block and cell death in liver cancer cells [10].

Thyroid transcription factor 1 (TTF1 NKX2-1) missense mutation has been implicated in thyroid tumorigenesis of familial non-medullary cancer [11], it is responsible for an increased proliferation rate of tumor cells in a rat tumor model of thyroid cancer [12]. Additionally, NKX2-1 has been found to be responsible for the down-regulation of the tumor suppressor gene ABI3 (ABI gene family member 3) [13].

In addition, long non-coding RNA H19, which is known for its metastatic property in several tumors [14], has also been found to be implicated in thyroid cancer [15]. Its ability to target miR-17-5p and to control the expression of genes related to cell viability, migration, and invasion has been shown in TPC1 and NIM thyroid cancer cells [14].

A successful treatment for thyroid cancer is strictly correlated with an active Sodium Iodide Symporter (SLC5A5; NIS), which allows the retention of radioiodine in the tumor cells, leading to cell death. Although the tyrosine kinase inhibitors (TKI), e.g., lenvatinib, sorafenib, and selumetinib, have shown progression-free survival in phase III clinical trials of patients affected by thyroid cancer [16], these compounds have shown strong adverse effects that are related to drug toxicity with frequent dose adjustments (dose interruption or reduction) and high therapy costs [17].

Histone deacetylase inhibitors (HDACi), e.g., Trichostatin A, panobinostat, and vorinostat (SAHA, Zolinza), are able to induce epigenetic modifications, leading to cell death and differentiation. In particular, they have been approved for the treatment of several solid and blood malignancies [18]. The restoration of NIS expression mediated by deacetylase inhibitors [19], as already shown in breast cancer cells (MDA 157 and MDA 468) [20,21], could represent a valuable advancement in the therapy of thyroid cancer. Clinical trials for the treatment of advanced thyroid cancers are showing promising results, e.g., in medullary thyroid cancer [22] and in combination with sorafenib [23].

Panobinostat has been shown to be effective at small doses in comparison with Trichostatin A and SAHA. Its use in therapy has shown moderate side effects. It is able to induce cell death by inducing alternative apoptotic mechanisms [24,25,26]. Furthermore, it is able to modulate the histone methylation [27] and the expression of miRNAs belonging to Let7 family, leading to down-regulation of their putative target HMGA2 and the miRNA cluster 19–31, then modulating the expression of key player of apoptosis and autophagy [28].

This study aimed to clarify the mechanisms induced by HDACi in in vitro models of thyroid cancer, highlighting the ability of these compounds to induce re-differentiation in thyroid cancer cell lines via the over-expression of NIS, the modulation of the long non-coding RNA H19, and the suppression of oncogenes, like TTF1 and HMGA2. Furthermore, the implications of hsa-let-7f-5p and hsa-let-7b-5p will be analyzed in order to understand their involvement in the regulation of their putative targets NIS and HMGA2 in our proposed model.

## 2. Materials and Methods

### 2.1. Cell Lines

Five cell lines with various histopathological background and mutation status were used. Two originated from papillary (TPC1 and BCPAP) [29], one from an anaplastic (C643) [30], one from a poorly differentiated (8505C) [31], and one from follicular (FTC133) [32] thyroid carcinoma. The mutation status is as follows: TPC1, Ret/PTC, C643, HRAS and TP53, BCPAP, BRAFV600E, 8505C, BRAFV600E and TP53, FTC133, and TP53 (Table 1).

TPC1; C643 and FTC133 cells were kindly provided by Prof. A. Zielke (Diakonie-Klinikum Stuttgart, Stuttgart, Germany); whereas BCPAP and 8505C cell lines were purchased from the DSMZ (Leibnitz Institute DSMZ-German Collection of Microorganisms and cell Cultures).

### 2.2. Cell Culture

All cell lines were grown in RPMI 1640 (Biochrom, Berlin, Germany) supplemented with 10% fetal bovine serum (FBS, Biochrom, Berlin, Germany) and 10 U/mL penicillin and 100 µg/mL streptomycin (Biochrom, Berlin, Germany) under standard conditions (37 °C; 5% CO_2_). They were routinely tested for Mycoplasma contamination.

### 2.3. Sample Collection

Patient sample collection and allowance to use for research purpose.

Tissue samples of patients with thyroid carcinoma, who underwent thyroid resection at the Philipps-University Hospital Marburg, were randomly collected and stored in liquid nitrogen tissue bank (−196 °C). 14 PTC, 8 FTC, and 12 ATC tissue samples were included in the study from patients resected between 1998 and 2012. Normal thyroid tissue from regions adjacent to the tumor area was collected as well and served as control tissue. Only two FTC and one ATC tissue samples were excluded from the study, because the RNA was not measurable. The study was conducted under the approval of the ethic committee of Marburg University Hospital (Nr. 166/09) and all of the patients provided signed informed consent.

### 2.4. Real-Time Cell *Via*bility Analysis

The xCELLigence RTCA SP system (Roche Applied Science; Mannheim, Germany) was used for real-time and time-dependent analysis of the cellular response of thyroid cancer cells following incubation with 1 nM to 10 µM panobinostat, 1 nM to 10 µM SAHA, and 1 nM to 10 µM Trichostatin A. To perform this analysis, 3000 cells were seeded in 150 μL complete growth medium per well in a 96-well E-plate (OLS; Bremen, Germany) and were incubated with the previously mentioned compounds. Cell Index, which indicates attachment and adherence of cells to the plate’s electrode, was continuously measured for the following 120 h using the cell impedance detection system. Data analysis was performed using the RTCA Software v1.2.1 for the calculation of the temporal dynamics of cellular attachment (i.e., viability).

### 2.5. RNA Isolation and Quantitative Real Time RT-PCR

Cells were seeded in 25 cm^2^ cell culture flasks (0.5 × 10^6^ cells/flask) and were treated with 10 nM panobinostat, 10 µM SAHA, and 1 µM Trichostatin A for 48 h. Total RNA, including short RNA was isolated by use of miRNeasy Mini Kit (Qiagen, Hilden, Germany), according to the manufacturer`s instruction.

miRNA enriched RNA was reverse transcribed with miScript II RT Kit (Qiagen, Hilden, Germany). cDNA was amplified with miScript SYBR Green PCR Kit (Qiagen, Hilden, Germany) using a CFX96 cycler (Bio-Rad, Munich, Germany), hsa-let-7f-5p (MS00006489), and hsa-let-7b-5p (MS00003122) miScript Primer Assays (Qiagen, Hilden, Germany). RNU6 (MS00029204) was amplified as internal control miRNA.

For the amplification of SLC5A5, HMGA2, NKX2-1, and H19 RNA lysates were reverse transcribed using the iScript cDNA Syntesis Kit (Bio-Rad, Munich, Germany). PCR was run with the SsoFast Eva Green Supermix (Bio-Rad, Munich, Germany) on CFX96 cycler. GAPDH was amplified as reference gene. Primer Assays for SLC5A5 (PPH10926A), NKX2-1 (QT00015981), lncRNA H19 (PPH05814B), and GAPDH (QT01192646) were purchased from Qiagen. HMGA2 (10025636) was purchased from Bio-Rad.

Data Analysis: Results were analyzed using CFX Manager (Bio-Rad, Munich, Germany) and Rest 2008. Significance was calculated using the t-test for paired samples. *p* < 0.05 was regarded as significant.

### 2.6. Protein Isolation and Western Blotting

Cells were seeded in 75 cm^2^ cell culture flasks (1.5 × 10^6^ cells/flask) and incubated with 10 nM panobinostat; 10 µM SAHA and 1 µM Trichostatin A for 48 h. The cell pellet was lysed with RIPA (Santa Cruz, Heidelberg, Germany) containing protease and phosphatase inhibitors (71µL 7× protease cocktail and 50 µL 10× phosphatase cocktail (Roche, Basel, Switzerland) per 500 µL RIPA buffer). Protein content was determined by BCA-assay (Pierce, Rockford, LA, USA). Samples that were adjusted to 50 µg were separated on SDS-PAGE (NuPAGE Novex 4–12% Bis-Tris gels, NuPage MOPS running buffer (Invitrogen by Life Technologies, Carlsbad, CA, USA)), and transferred to nitrocellulose (Amersham, Piscataway, NJ, USA). Membranes were probed using anti-human sodium iodide symporter (hNIS); clone FP5A (1:500) (Thermofisher Scientific, Fremont, CA, USA) as primary antibody. HRP conjugated secondary antibodies were from SIGMA-Aldrich. Visualization was performed by ECL western blotting reagent (Amersham, Piscataway, NJ, USA) and using an image capture and analysis system (Fusion; PeqLab, Erlangen, Germany). Equal loading was verified by anti-GAPDH (ab 9485. 1:2500) (Abcam, Cambridge, MA, USA).

### 2.7. In Vitro Radioiodine Up-Take

For the analysis of radioiodine up-take, the cells were seeded in 6-well plates at a density of 4 × 10^5^ cells/well. Immediately after treatment with 10 nM panobinostat, 10 µM SAHA and 1 µM Trichostatin A; 1 Mbq I^131^ was added to the cells and the plates were incubated for 24 and 48 h in complete growth medium (see Cell Culture) containing 0.4 × 10^−3^ mmol/L Magnesium Sulfate (0.5 % elemental Magnesium). Subsequently, the cells were washed with PBS and trypsinized for 5 min. The suspension was rinsed with 3 mL PBS, collected, and centrifuged at 900 rpm for 5 min. The supernatant was discarded and the pellet was suspended in 5 mL PBS. The suspension was once more centrifuged. The supernatant was discarded and the cells were processed into a Gamma counter for the measurement of the retained radioactive I^131^. The untreated cells were used as control for the measurement.

### 2.8. Statistical Analysis

Data were collected using Excel (Microsoft Office, Microsoft Corporation, Redmond, WA, USA). Significance was calculated using the t-test for paired samples. *p* < 0.05 was regarded as significant.

## 3. Results

### 3.1. Cytotoxic Effects of Deacetylase Inhibitors in Thyroid Cancer Cells

**Figure 1:**
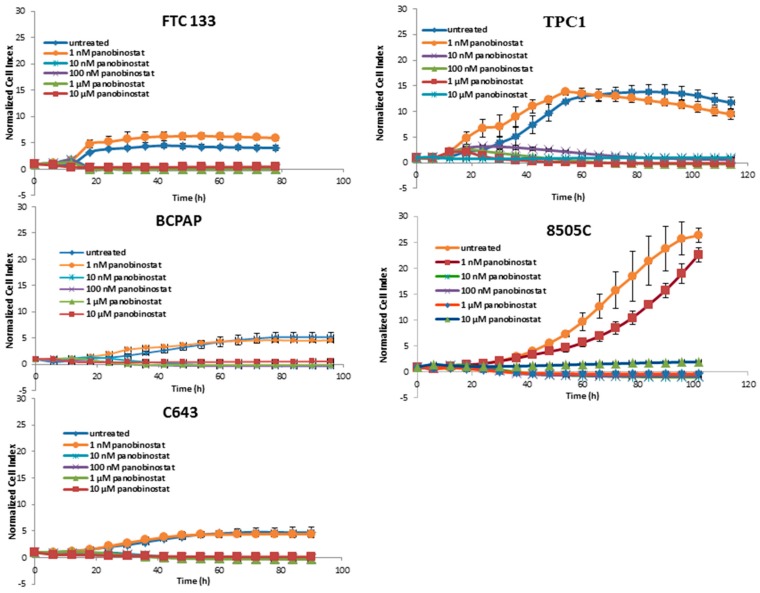
Real time cell viability of thyroid cancer cells treated with panobinostat. FTC133, TPC1, BCPAP, 8505C, and C643 cells were plated on gold-coated 96-well plates. After approx. 24 h, panobinostat was added at concentration between 1 nM and 10 µM. The cell viability was monitored constantly for up to 100 h. Cell index was normalized to the time point of treatment. Shown are means ± SD of three independent experiments performed in triplicates.

As shown in Figure 1, a significant reduction of cell viability could be observed after treatment with 10 nM panobinostat.

**Figure 2:**
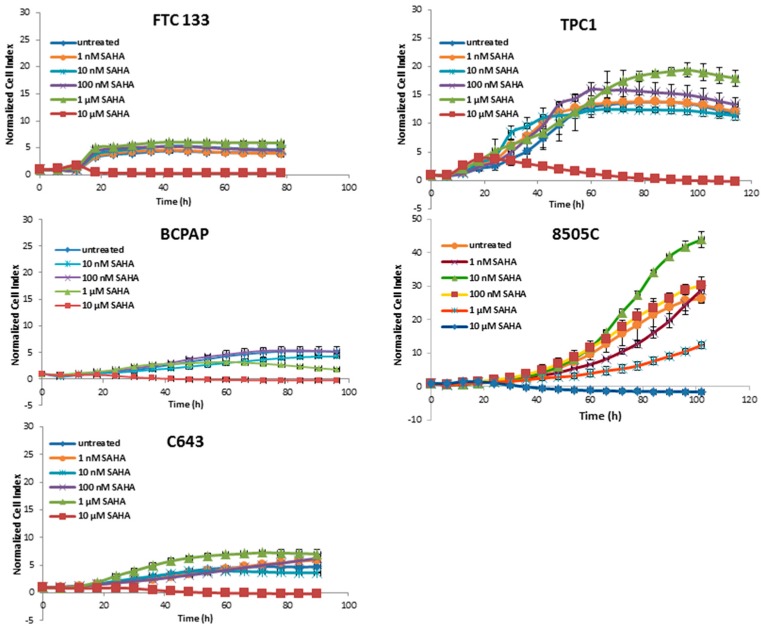
Real time cell viability of thyroid cancer cells treated with SAHA. FTC133, TPC1, BCPAP, 8505C, and C643 cells were plated on gold-coated 96-well plates. After approx. 24 h, SAHA was added at concentration between 1 nM and 10 µM. The cell viability was monitored constantly for up to 100 h. Cell index was normalized to the time point of treatment. Shown are means ± SD of three independent experiments performed in triplicates.

The treatment with SAHA was almost ineffective at most concentrations; only at 10 µM a cytotoxic effect was present (Figure 2).

**Figure 3:**
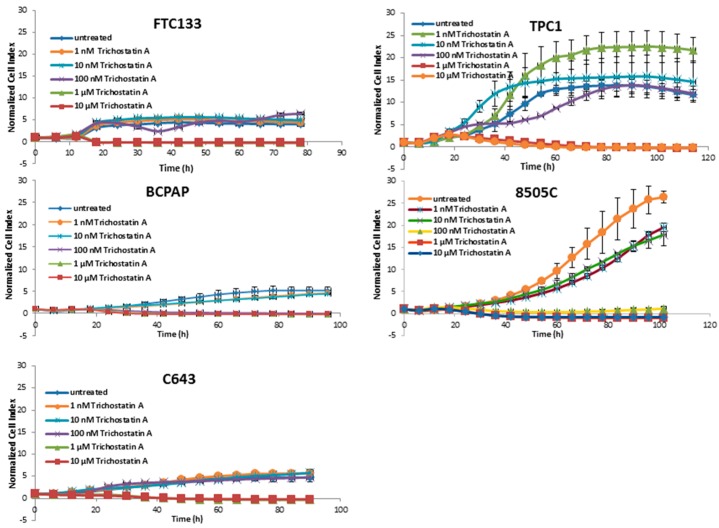
Real time cell viability of thyroid cancer cells treated with Trichostatin A. FTC133, TPC1, BCPAP, 8505C, and C643 cells were plated on gold-coated 96-well plates. After approx. 24 h, Trichostatin A was added at concentration between 1 nM and 10 µM. The cell viability was monitored constantly for up to 100 h. Cell index was normalized to the time point of treatment. Shown are means ± SD of three independent experiments performed in triplicates.

Trichostatin A affected the cell viability of all cell lines beginning at 1 µM (Figure 3).

Panobinostat showed the strongest efficacy in comparison with SAHA and Trichostatin A by showing a reduction of cell viability after treatment with 10 nM. Thus, 10 nM panobinostat, 10 µM SAHA, and 1 µM Trichostatin were employed as the lowest best efficacious concentrations for the following experiments.

### 3.2. Expression of SLC5A5 (NIS), HMGA2, and Their Regulatory miRNAs Hsa-let-7b-5p Hsa-let-7f-5p 

**Figure 4:**
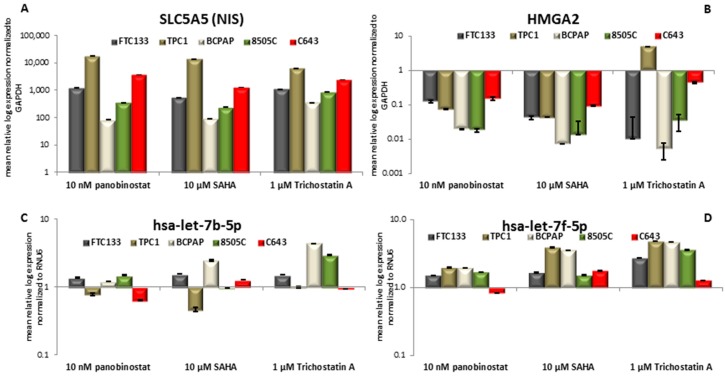
NIS; HMGA2; hsa-let-7b-5p and hsa-let-7f-5p RNA level modulation. RT-qPCR analysis of NIS (**A**); HMGA2 (**B**); hsa-let-7b-5p (**C**); and, hsa-let-7f-5p (**D**) in FTC133; TPC1; BCPAP; 8505C; and, C643 cells after 48h of treatment with 10 nM panobinostat; 10 µM SAHA; and, 1 µM Trichostatin A. mRNA expression was normalized to GAPDH, miRNA expression was normalized to RNU6. Results are expressed relative to untreated controls set at 1.0. Shown are means ± SEM of three independent experiments performed in triplicates.

The cell lines that were included in the study showed almost no detectable expression of the SLC5A5 transcript level. In the conducted experiments, 48h treatment with pan-deacetylase inhibitors panobinostat (10 nM), SAHA (10 µM), and Trichostatin A (1 µM) induced a significant restoration/over-expression of SLC5A5 in all the cell lines (Figure 4A).

It is of note that 48 h treatment with HDACi caused a significant down-regulation of HMGA2 transcript in all of the cell lines (Figure 4B). The negative regulation of HMGA2 was independent of the pathological characterization of the cell lines.

Furthermore, the status of two members of Let7 miRNAs family, hsa-let-7b-5p, and hsa-let-7f-5p, was evaluated because of their putative role as binder and repressor of SLC5A5 and HMGA2 transcripts. Hsa-let-7b-5p (Figure 4C) showed a stable expression after 48 h treatment with 10 nM panobinostat; 10 µM SAHA caused a significant down-regulation of hsa-let-7b-5p in TPC1 cells and a significant over-expression of it in BCPAP cells. 48 h treatment with 1 µM Trichostatin A caused an over-expression of hsa-let-7b-5p in BCPAP and 8505C cells. Hsa-let-7f-5p (Figure 4D) was not significantly over-expressed in four cell lines that were treated for 48 h with 10 nM panobinostat, C643 showed only a not significant suppression of it. 10 µM SAHA caused an over-expression of hsa-let-7f-5p in TPC1 and BCPAP cells; FTC133, 8505C, and C643 showed a stable expression of it. The treatment with 1 µM Trichostatin A (48 h) caused a significant over-expression of hsa-let-7f-5p in four cell lines; C643 cells showed a stable expression of hsa-let-7f-5p. The stable and/or over-expressed level of hsa-let-7b-5p and hsa-let-7f-5p could inversely correlate with the down-regulation of HMGA2 transcript only. SLC5A5 transcript expression resulted independent from miRNAs expression.

### 3.3. NIS Protein Modulation after Treatment with HDACi

**Figure 5:**
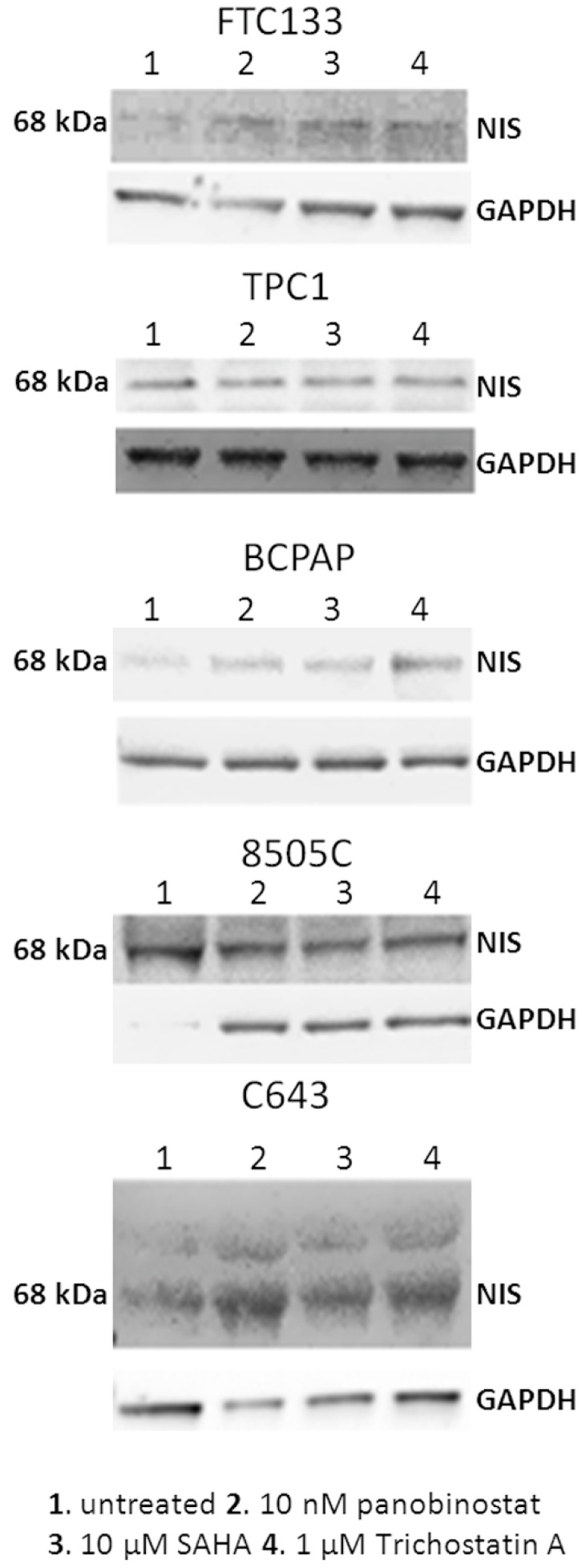
NIS protein level after treatment with deacetylase inhibitors. NIS protein level was detected in FTC133, TPC1, BCPAP, 8505C, and C643 cells after 48 h of treatment with 10 nM panobinostat, 10 µM SAHA, and 1 µM Trichostatin A. The band detected a protein of 68 kDa corresponding to NIS. GAPDH was detected as loading control.

In order to determine whether HDACi caused change in protein level of NIS, all of the thyroid cancer cell lines that are included in this study were incubated for 48 h with 10 nM panobinostat, 10 µM SAHA, and 1 µM Trichostatin A, and whole cell lysate was processed by western blot. As shown in Figure 5, panobinostat, SAHA, and Trichostatin A caused a strong re-expression of NIS protein level in FTC133, BCPAP, and C643 cells in comparison with untreated cells. In TPC1 and 8505C cells, the expression of NIS was stable after treatment with HDACi in comparison with untreated cells.

Interestingly, HDACi caused a re-expression and/or a strong over-expression of NIS protein in cells lacking of NIS protein or expressing it at very low level, whereas no effect was observed in cell lines showing a basal level of NIS protein.

### 3.4. Radioiodine Up-take after HDACi Treatment

**Figure 6:**
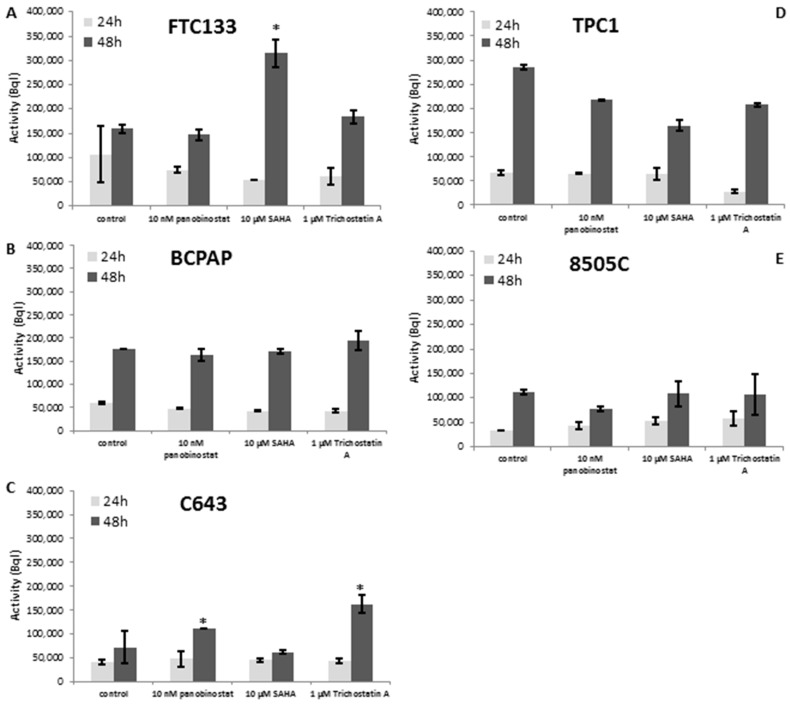
Radioiodine activity measurement after treatment with deacetylase inhibitors. FTC133 (**A**), BCPAP (**B**), C643 (**C**), TPC1 (**D**) and 8505C (**E**) cells were treated for 24 and 48 h with 10 nM panobinostat, 10 µM SAHA and 1 µM Trichostatin A and 1 MBql I^131^. The radioactivity of cells was measured on a gamma counter. Data represent mean ± SEM of experiments performed in triplicates.* *p* < 0.05 regarded as significant change between treated and untreated control samples.

The function of NIS protein was analyzed by radioiodine up-take measurement after HDACi treatment. The activity of NIS was almost stable in all five cell lines. Interestingly, 48 h treatment with 10 µM SAHA and 1 µM Trichostatin A caused an increase of radioiodine up-take in FTC133 cells (Figure 6A). C643 cells showed a higher iodine activity after 48 h treatment with 10 nM panobinostat and 1 µM Trichostatin A (Figure 6C).

### 3.5. TTF1 Expression in Primary Thyroid Tumor Samples and in Vitro after Treatment with HDACi

**Figure 7:**
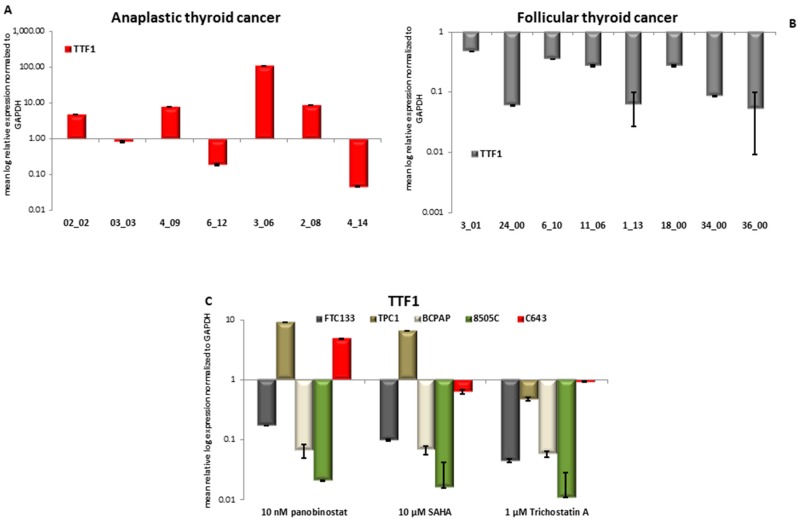
Expression of TTF1 in anaplastic thyroid cancer (ATC), Follicular thyroid cancer (FTC), and thyroid cancer cells treated with deacetylase inhibitors. RT-qPCR analysis of TTF1 transcript level in ATC (**A**), FTC (**B**) and FTC133, TPC1, BCPAP, 8505C, and C643 cells (**C**) after 48 h of treatment with 10 nM panobinostat, 10 µM SAHA and 1 µM Trichostatin A. mRNA expression was normalized to GAPDH. Results are expressed relative to untreated controls set at 1.0. Shown are means ± SEM of three independent experiments performed in triplicates.

First, the expression of TTF1 was determined in thirteen samples of patients affected by ATC, eight samples of FTC, and 16 samples of PTC. Seven patients out of 13 ATC showed a detectable expression of TTF1. In particular, it was observed a significant over-expression (*p* < 0.05) of TTF1 in four samples, two samples showed a significantly lower expression (*p* < 0.05), and one sample showed no variation (Figure 7A), in comparison with normal thyroid tissue from regions, adjacent to the tumor area, used as control. All eight FTC samples showed a detectable significantly (*p* < 0.05) lower expression of TTF1 (Figure 7B) when compared to normal thyroid tissue. The analysis of PTC samples showed no detectable expression of TTF1 transcript. Figure 7 panel C shows the analysis of TTF1 transcript level in five thyroid cancer cell lines treated for 48 h with 10 nM panobinostat, 10 µM SAHA, and 1 µM Trichostatin A. The level of TTF1 was significantly down regulated in FTC133, BCPAP, and 8505C cells treated with 10 nM panobinostat; whereas, in TPC1 and C643 cells, an up-regulation of TTF1 transcript was observed. The treatment with 10 µM SAHA caused a down-regulation of TTF1 in FTC133, BCPAP, and 8505C cells; also, TPC1 cells showed an up-regulation and C643 cells a stable expression of TTF1. 1 µM Trichostatin caused a significant down-regulation of TTF1 transcript in FTC133, TPC1, BCPAP, and 8505C cells; the level of TTF1 transcript was stable in C643 cells.

In conclusion, the transcript level of TTF1 was strongly suppressed in all of the cell lines treated with HDACi. C643 cells isolated from anaplastic thyroid cancer and having a highly undifferentiated status showed a high or basal expression of TTF1, even after treatment with HDACi.

### 3.6. Expression of Long Non-Coding RNA H19 in Primary Thyroid Tumor Samples and In Vitro after Treatment with HDACi

**Figure 8:**
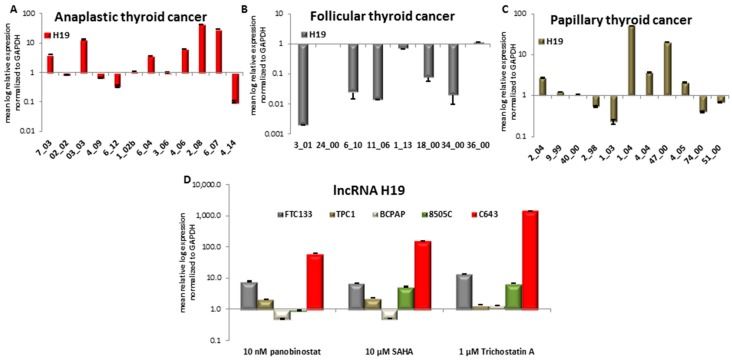
Expression of lncRNA H19 in ATC, FTC, PTC, and thyroid cancer cells that were treated with deacetylase inhibitors. RT-qPCR analysis of TTF1 transcript level in ATC (**A**), FTC (**B**), PTC (**C**) and FTC133, TPC1, BCPAP, 8505C and C643 cells (**D**) after 48 h of treatment with 10 nM panobinostat, 10 µM SAHA and 1 µM Trichostatin A. mRNA expression was normalized to GAPDH. Results are expressed relative to untreated controls set at 1.0. Shown are means ± SEM of three independent experiments performed in triplicates.

First, we investigated the expression of H19 in samples of patients that were affected by ATC, FTC, and PTC. Six ATC samples showed an over-expression of H19, two of them a down-regulation, and four showed a stable expression in comparison with normal thyroid tissue used as control (Figure 8A). Analysis of eight FTC samples determined that five patients had a down-regulated H19 and the other three patients showed a stable expression of it (Figure 8B). Only 11 out of 16 PTC samples showed an expression of H19. In detail, five samples showed a significant over-expression, two had a significant down-regulation, whereas the other four samples showed no variation of H19 expression (Figure 8C).

The expression of H19 was found heterogeneous in all three distinct tumor entities and no correlation could be attributed between tumor type and H19 expression.

We therefore determined H19 expression after 48 h treatment with HDACi in FTC133, TPC1, BCPAP, 8505C, and C643 TC cell lines (Figure 8D).

H19 showed a nearly 10-fold increase in FTC133 cells treated for 48 h with 10 nM panobinostat, 10 µM SAHA, and 1 µM Trichostatin A. In the PDTC cell line 8505C, a 5-10-fold increase was seen only after treatment with SAHA and Trichostatin A. In C643 cells, a significant increase was observed with a more than 100-fold increase of H19 level after panobinostat and SAHA, respectively. Treatment with 1 µM Trichostatin A caused an H19 increase of more than 1000-fold. BCPAP and TPC1 cells both representing PTC showed a basal expression of H19 after treatment with HDACi, in comparison with untreated cells that were used as control.

## 4. Discussion

Differentiated thyroid cancer has a good prognosis, but certain poorly differentiated TC and anaplastic TC have a very different malignant behavior. They show fast tumor growth, early metastases, and scarce or totally absent radioiodine up-take. Therapy remains challenging and new drugs have emerged, but their efficacy is still not well defined.

In the present study, the cytotoxic effects of histone deacetylase inhibitors panobinostat, SAHA, and Trichostatin A were tested in five thyroid cancer cell lines. Interestingly, all three deacetylase inhibitors showed a strong cytotoxic effect in all of the tested cell lines independently of their tumor type and origin. In particular, the three compounds showed the same efficacy in a time and dose dependent manner in cell lines deriving from ATC, FTC, and PTC. Panobinostat, in particular, showed the highest efficacy, already at 10 nM. Here, we could confirm, for the first time, the highest potency of panobinostat in comparison with other pan-deacetylase inhibitors, e.g., SAHA and Trichostatin A. Thus, confirming previous studies showing the higher efficacy of panobinostat for the treatment of other solid tumors and its capability to overcome the resistance to SAHA in cutaneous T-cell lymphoma [33,34,35].

It is widely accepted that histone deacetylase inhibitors can induce re-expression of tumor suppressor silenced genes in cancer cells [36], and that they can induce radioiodine up-take in patients affected by radioiodine refractory thyroid cancer [19]. In our study, the expression of NIS at the transcript and protein level was examined. Five thyroid cancer cell lines showed a strong re-expression/over-expression of NIS transcript after treatment with deacetylase inhibitors. Additionally, the protein level of NIS was up-regulated in three cell lines; FTC133, BCPAP and C643, the ones that showed a low or absent basal level of NIS protein. These data, including the treatment with nanomolar concentration of panobinostat, confirmed the previous study of Furuya and colleagues showing the re-expression of SLC5A5 (NIS), TPO (thyroperoxidase), and Tg (Thyroglobulin) mRNA after treatment with HDACi depsipeptide and Trichostatin A in poorly differentiated and anaplastic thyroid cancer in vitro and in vivo [37].

This study could confirm that treatment with deacetylase inhibitors is able to increase the radioiodine up-take in thyroid cancer cells, as previously shown [37]. However, this effect was observed in only two of the cell lines that are involved in the study, the poorly differentiated cell line FTC 133 and anaplastic cell line C643, whose showed an increase of I^131^ retention after treatment with deacetylase inhibitors. Interestingly, those cells were characterized by an up-regulation of NIS protein level. So, we could assume that the cell lines expressing low or absent NIS were the most sensitive to deacetylase inhibitors modulation of radioiodine up-take. Papillary thyroid cancer cells TPC-1 and the poorly differentiated cells 8505C did not show any difference in radioiodine up-take after HDACi treatment, as already observed for the NIS protein level. BCPAP cells, despite an increase of NIS protein level after treatment, mainly with 10 nM Trichostatin A, showed also no increase of radioiodine up-take.

Based on previous studies describing the localization of functional NIS protein at the baso-lateral cell membrane [38] we could assume that the treatment with deacetylase inhibitors could not only restore the expression of NIS in thyroid cancer cells, but also modulate the activity of the sodium iodide symporter, as confirmed by the partial increase of radioiodine up-take. As previously reported, NIS expression could be modulated by the expression of miRNAs belonging to Let7 family. In particular, in silico analysis reported NIS transcript as validated target of hsa-let-7f-5p [6]. The treatment with deacetylase inhibitors caused a stable or not significant over-expression of hsa-let-7f-5p in thyroid cancer cells that could exclude its role as NIS’ trap in our proposed model. Furthermore, hsa-let-7b-5p was detected. Except for a significant down-regulation in TPC1 cells treated with 10 µM SAHA, it was observed a stable expression of this miRNA in thyroid cancer cells that were treated with deacetylase inhibitors. Interestingly, the stable expression of those miRNAs belonging to Let7 family could be inversely correlated with the significant down-regulation of HMGA2, which is a non-transcription factor with well-known oncogenic properties. These results confirm previous studies that were conducted in liver cancer cells treated with panobinostat [10].

Furthermore, this study focused on the role exerted by H19 in thyroid cancer cells that were treated with deacetylase inhibitors. H19, a 2.7 kb gene transcribed from the maternally inherited allele, is a long non-coding RNA that is highly expressed during tumorigenesis [39]. Lin Liu and colleagues demonstrated that H19 is able to bind and block miRNA hsa-miR-17-5p, leading to the modulation of YES1 translation in thyroid carcinogenesis [14], thus promoting in vitro cell proliferation, migration, and invasion. In hepatocellular carcinoma, H19 was shown to act as a tumor suppressor [40]. 

In this study, it was observed that H19 is present in anaplastic, follicular and papillary thyroid tumors and it is heterogeneously expressed. Specifically, ATC showed a general over-expression of H19, whereas a general down-regulation was observed in FTC. Papillary thyroid cancer samples showed a heterogeneous expression of H19. Interestingly, despite the tumor type, all five tested cell lines that were treated with HDACi showed a stable and/or significant over-expressed level of H19 transcript. As previously published, acetylation of histones promotes the over-expression of H19, together with Insulin Growth Factor 2(IGF2) [41]. Furthermore, H19 has been shown to inhibit Insulin receptor Substrate 1(IRS-1), leading to reduction of cell viability, migration, and invasion in thyroid cancer cells [42]. These studies further confirm the general mechanism of induction of H19 in our proposed model, thus supporting an alternative tumor suppressor role exerted by H19 in thyroid cancer cells that are treated with deacetylase inhibitors, leading to differentiation and cell death. 

Nonetheless, this study showed that TTF1, which is a well-known thyroid transcription factor with oncogenic property, is highly expressed in ATC, suppressed in FTC, and not detectable in PTC samples. Treatment with deacetylase inhibitors panobinostat, SAHA, and Trichostatin A caused a significant down-regulation of the TTF1 transcript in FTC133, BCPAP, and 8505C cells. TPC1 and C643 cells, which are the most undifferentiated cells, showed a high expression or similar to untreated cells, even after treatment. The suppression of TTF1 could favor the re-expression of the tumor suppressor ABI3, as previously published [13], thus supporting the cell death mechanism mediated by deacetylase inhibitors in thyroid cancer cells.

## 5. Conclusions

To summarize, treatment with the deacetylase inhibitors panobinostat, SAHA, and Trichostatin A was effective in inducing cell death in thyroid cancer cells. The mechanism was independent of tumor origin and its mutation status. Interestingly, panobinostat was the compound that had the highest efficacy with the lowest concentration (10 nM), avoiding severe side effects that have been reported for SAHA and Trichostatin A in solid and hematologic cancer [43]. Treatment with deacetylase inhibitors triggered the well-defined molecular mechanisms that involved the re-expression of NIS, the up-regulation of H19, and the suppression of the oncogenes TTF1 and HMGA2. This represents a favorable aspect for the clinical application of deacetylase inhibitors in patients with radioiodine refractory thyroid cancer that needs to be evaluated in future studies.

## Figures and Tables

**Table 1:** Schematic representation of cell lines used in the study. Origin specification and mutation panel.

	FTC133	TPC1	BCPAP	8505C	C643
Origin	follicular	papillary	papillary	poorly differentiated	anaplastic
TP53	mut			mut	mut
HRAS					mut
Ret/PTC		mut			
BRAF			mut; V600	mut; V600	
